# Systemic characterization of alternative splicing related to prognosis and immune infiltration in malignant mesothelioma

**DOI:** 10.1186/s12885-021-08548-3

**Published:** 2021-07-22

**Authors:** Jinzhi Lai, Hainan Yang, Tianwen Xu

**Affiliations:** 1 Department of Oncology, The Second Affiliated Hospital of Fujian Medical University, Quanzhou, 362000 Fujian China; 2 Department of Ultrasound, The Second Affiliated Hospital of Fujian Medical University, Quanzhou, Fujian China

**Keywords:** Malignant mesothelioma, Alternative splicing, Prognostic signature, Risk score, Immune infiltration

## Abstract

**Background:**

Malignant mesothelioma (MM) is a relatively rare and highly lethal tumor with few treatment options. Thus, it is important to identify prognostic markers that can help clinicians diagnose mesothelioma earlier and assess disease activity more accurately. Alternative splicing (AS) events have been recognized as critical signatures for tumor diagnosis and treatment in multiple cancers, including MM.

**Methods:**

We systematically examined the AS events and clinical information of 83 MM samples from TCGA database. Univariate Cox regression analysis was used to identify AS events associated with overall survival. LASSO analyses followed by multivariate Cox regression analyses were conducted to construct the prognostic signatures and assess the accuracy of these prognostic signatures by receiver operating characteristic (ROC) curve and Kaplan–Meier survival analyses. The ImmuCellAI and ssGSEA algorithms were used to assess the degrees of immune cell infiltration in MM samples. The survival-related splicing regulatory network was established based on the correlation between survival-related AS events and splicing factors (SFs).

**Results:**

A total of 3976 AS events associated with overall survival were identified by univariate Cox regression analysis, and ES events accounted for the greatest proportion. We constructed prognostic signatures based on survival-related AS events. The prognostic signatures proved to be an efficient predictor with an area under the curve (AUC) greater than 0.9. Additionally, the risk score based on 6 key AS events proved to be an independent prognostic factor, and a nomogram composed of 6 key AS events was established. We found that the risk score was significantly decreased in patients with the epithelioid subtype. In addition, unsupervised clustering clearly showed that the risk score was associated with immune cell infiltration. The abundances of cytotoxic T (Tc) cells, natural killer (NK) cells and T-helper 17 (Th17) cells were higher in the high-risk group, whereas the abundances of induced regulatory T (iTreg) cells were lower in the high-risk group. Finally, we identified 3 SFs (HSPB1, INTS1 and LUC7L2) that were significantly associated with MM patient survival and then constructed a regulatory network between the 3 SFs and survival-related AS to reveal potential regulatory mechanisms in MM.

**Conclusion:**

Our study provided a prognostic signature based on 6 key events, representing a better effective tumor-specific diagnostic and prognostic marker than the TNM staging system. AS events that are correlated with the immune system may be potential therapeutic targets for MM.

**Supplementary Information:**

The online version contains supplementary material available at 10.1186/s12885-021-08548-3.

## Background

Malignant mesothelioma (MM) is a rare and aggressive kind of cancer that originates in the mesothelial surfaces of the pleura and other sites [1, 2]. Approximately 3000 new cases and 3000 deaths due to MM occur annually in the United States, and approximately 80–90% of mesothelioma cases are connected to asbestos exposure [3]. MM is a heterogeneous tumor that includes the following three major histologic subtypes: epithelioid, sarcomatoid and biphasic. The epithelioid subtype is the most common type of mesothelioma, and it has a better prognosis than the other subtypes [4]. The median overall survival is approximately 1 year in patients with advanced surgically unresectable mesothelioma, and the 5-year overall survival (OS) is approximately 10% [5]. Although surgical resection is effective in patients with early-stage disease, most patients are diagnosed at advanced stages, in which traditional drug regimens are ineffective, and for these patients, a cure is not possible [6]. Thus, the early diagnosis of mesothelioma is an important factor in prognosis and treatment options. Evidence has emerged that the prevalence of MM is attributable to inherited mutations of susceptibility genes [7]. However, the molecular mechanisms of MM are still largely unknown, and the relevance of susceptibility genes to carcinogenesis also remains mostly unknown.

Alternative splicing is a molecular splicing process by which exons or noncoding regions are differentially joined together or skipped to produce different mature mRNAs from a single gene during transcription [8]. The majority of human genes undergo alternative splicing to produce multiple mRNA isoforms, which plays a crucial role in generating protein diversity and provides an opportunity for gene/protein regulation [9], and alternative splicing also has an essential role in cellular differentiation and organism development [10, 11]. AS events can be classified into seven types according to the combinational types of splice sites, including alternate donor (AD), alternate acceptor (AA), alternate terminator (AT), alternate promoter (AP), mutually exclusive exons (ME), exon skip (ES), and retained intron (RI) [12]. In addition to increasing the diversity and functional capacity of a gene during posttranscriptional processing, AS is often associated with the occurrence of cancer driver mutations in encoded genes [13]. Recently, high-throughput sequencing technologies have revealed that AS is involved in multiple pathologies of cancer [14–16]. Cancer cells generate abnormal proteins with missing, altered, or inserted domains, resulting in oncogenesis [14]. Changes in alternative splicing may recapitulate cancer-related phenotypes by inducing cell proliferation or avoiding apoptosis [17]. Therefore, AS changes are recognized as important signatures of tumor progression, diagnosis and treatment [18, 19]. Currently, the analysis of tumor AS is a promising step forward in providing potential sources for diagnostic, prognostic and therapeutic strategies [20]. However, the relationship between AS and the prognosis of MM has not been extensively studied.

AS events have recently been identified as a source of tumor-specific neoantigens and play a critical role in the formation of the tumor microenvironment [21, 22]. Accumulating evidence has shown that AS has potential targets for immunotherapy, but it remains unknown how AS affects the immune system of MM patients and whether AS could be a target for immunotherapy. Here, we performed a genome-wide analysis of AS profiling in MM, providing an overall view of survival-related AS events in MM patients. A total of 3976 AS events were identified as candidate survival-related AS events. We established prognostic signatures based on survival-related AS events for MM patients, which were proved to be an efficient predictor for survival. We further demonstrated that the prognostic signature composed of 6 AS events was associated with the infiltration of immune cells in MM patients. Finally, a potential regulatory network was constructed to characterize the associations between splicing factors and AS. Understanding the AS events that could drive MM is crucial for the successful development of diagnostic and therapeutic modalities. Our study may contribute to understanding the mechanisms underlying the progression of MM and may shed new light on developing potential therapeutic targets in the future.

## Methods

### Data acquisition

The mRNA data and clinical information of MM patients were obtained from TCGA (https://portal.gdc.cancer.gov/, accessed February 1, 2021), and 87 specimens of primary MM were included in this study. The percent spliced in (PSI) values of AS events in MM samples were downloaded from TCGA SpliceSeq (https://bioinformatics.mdanderson.org/TCGASpliceSeq, February April 1, 2021), a web-based resource that has been widely used to explore the AS patterns of TCGA tumors [23]. These AS events can be classified into seven types, including the alternate donor (AD), alternate acceptor (AA), alternate terminator (AT), alternate promoter (AP), mutually exclusive exon (ME), exon skip (ES) and retained intron (RI).

### Identification of survival-related AS events and functional enrichment analysis

To identify survival-related AS events, univariate Cox regression analysis was performed to evaluate the association between AS events and the overall survival time of MM patients. AS events expressing significant *p* values < 0.05 were selected as survival-related AS events. UpSet plots were used to display the survival-related AS events based on the seven types of AS events. Moreover, bubble charts were used to summarize the top 10 AS events. The parent genes of the survival-related AS events were subjected to functional enrichment analysis using the ClusterProfiler R package [24]. Terms with adjusted *p*-values < 0.05 were considered significantly enriched. The top 20 significant terms in the Kyoto Encyclopedia of Genes and Genomes (KEGG) pathways and Gene Ontology (GO) categories, including the molecular function (MF), biological process (BP) and cellular component (CC) categories, were visualized with bubble diagrams [25–27].

### Multivariate prognostic model constructed by LASSO regression

Least absolute shrinkage and selection operator (LASSO) regression was performed to minimize overfitting and identify the most significant survival-related AS events. After testing for collinearity, stepwise multivariate Cox regression analysis was performed to calculate the prognostic risk scores for OS prediction based on the seven types of AS events. A multivariate prognostic model was constructed based on the PSI value of the AS events. The risk score was calculated using the following formula:

$$ \mathrm{Risk}\kern0.17em \mathrm{score}=\sum \limits_{i=1}^n\beta i\times \mathrm{PSI} $$

where n, β*i*, and PSI represent the number of survival-related AS events, the coefficient index of the AS events, and the PSI value of the AS events, respectively. According to the median risk score, the samples were divided into a high-risk group and a low-risk group. Kaplan-Meier survival curves and the log-rank test were used to analyze the survival rates between the low-risk and high-risk groups. A receiver operating characteristic (ROC) curve was constructed, and the area under the curve (AUC) was calculated using the “survival ROC” package in R. The expression heatmap, distribution of risk scores and survival times related to the signature were displayed. Finally, to limit overfitting, we used a resampling technique to estimate model accuracy. The C-index was the indicator for assaying the performance of the prognostic model, and we calculated the C-index through bootstrap resampling to estimate model accuracy using the “dplyr”, “rms”, “survival” and “pec” R packages.

### Independence of the prognostic signature

The following clinicopathological parameters from TCGA database, including asbestos exposure, age, gender, T stage, N stage, M stage and clinical stage were used for further analysis. Univariate and multivariate Cox regression analyses were applied to evaluate whether the AS event-based prognostic risk score was an independent risk factor for survival.

### Analysis of immune cell infiltration level and tumor-infiltrating immune cells in MM patients

We assessed the enrichment levels of the 29 immune signatures in each MM sample by the single-sample gene-set enrichment analysis (ssGSEA) score. These immune signatures for immune cell types were downloaded from Bindea et al. [28]. According to the enrichment levels (ssGSEA scores) of the 29 immune signatures, tumors with qualitatively different immune cell infiltration subtypes were grouped using hierarchical clustering.

Immune Cell Abundance Identifier (ImmuCellAI) was performed to calculate the relative abundances of 24 types of immune cells from transcriptome data [29]. These immune cells are comprised of B cells, macrophages, monocytes, neutrophils, DCs, NK cells and 18 T cell subtypes (CD4 T cells; CD8 T cells; naïve CD4 T cells; naïve CD8 T cells; cytotoxic T (Tc) cells; exhausted T (Tex) cells; type 1 regulatory T (Tr1) cells; natural regulatory T (nTreg) cells; induced regulatory T (iTreg) cells; T-helper 1, 2 and 17 (Th1, Th2 and Th17) cells; follicular T-helper (Tfh) cells; central memory T (Tcm) cells; effector memory T (Tem) cells; natural killer T (NKT) cells; mucosal-associated invariant T (MAIT) cells; and gamma-delta T cells).

### SF-AS regulatory network

A list of 119 SFs was acquired from a previous study that analyzed whole-exome sequencing data across 33 tumor types [30]. The expression profiles of SF genes were obtained from TCGA database. Subsequent univariate Cox regression analysis was conducted to identify survival-associated SFs in the MM samples. To estimate the correlation between AS events and SFs, a correlation network between the survival-associated SFs and survival-related AS events was constructed based on the Spearman’s test. *P* values less than 0.05 and Pearson’s correlation coefficients greater than 0.4 were considered statistically significant. The potential SF-AS regulatory network was visualized by Cytoscape software (version 3.7.2).

### Statistical analysis and R package

All statistical analyses were conducted using R (version 3.6.3). The intersections and aggregates of different types of AS events were assessed using the “UpSetR” package. KEGG and GO analyses were performed using the “ClusterProfiler” package. Survival analysis was performed using the “survival” and “survivalROC” packages. LASSO multivariate Cox analysis was performed using the “glmnet” package. Kaplan–Meier curves and the log-rank test were used to evaluate the statistical significance of the survival rates. The predictive accuracy of the prognostic signature was determined by ROC curve analysis.

## Results

### Analysis of AS events in MM patients

**Fig. 1:**
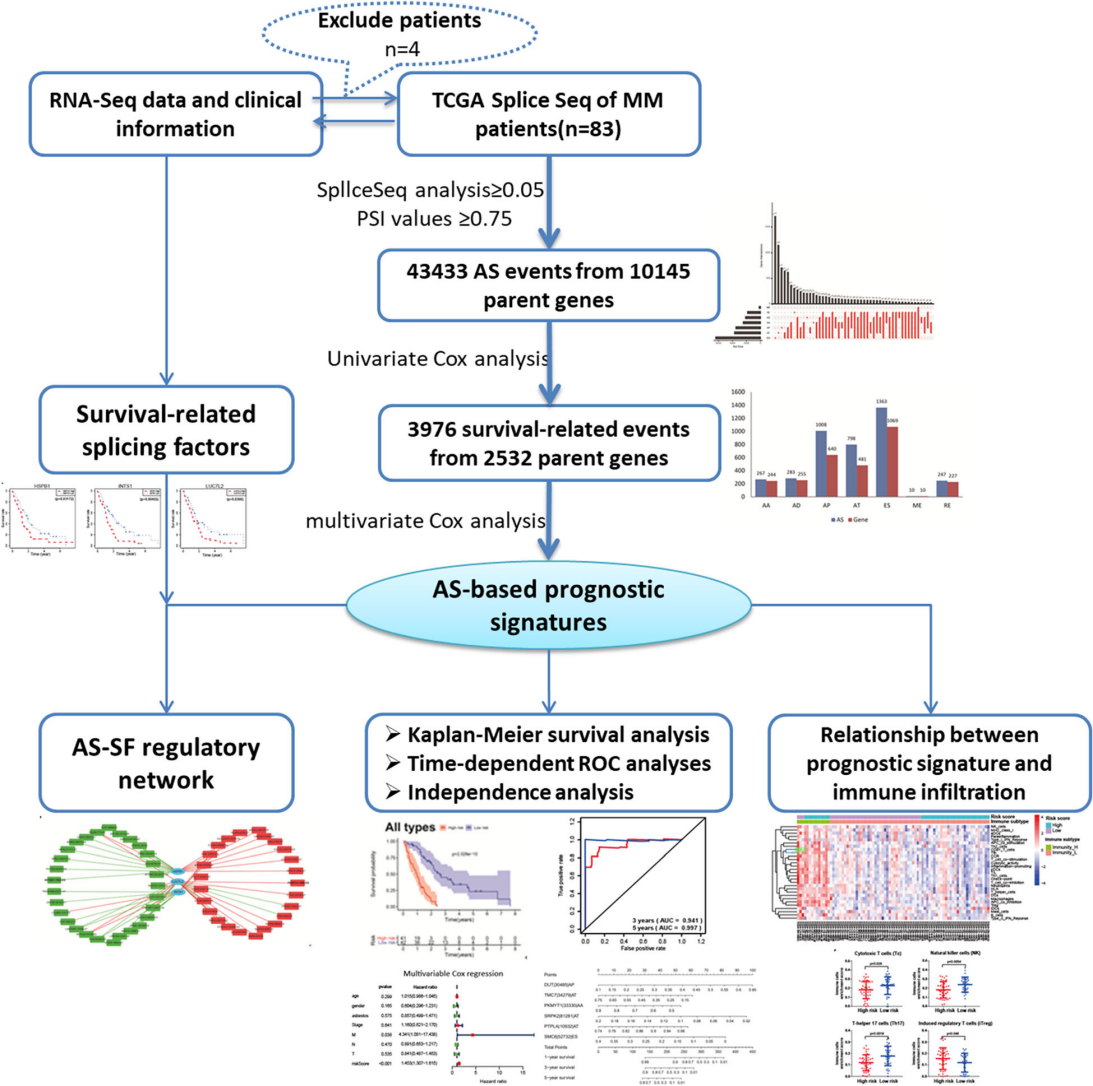
Experimental design and analyses presented in our work

The workflow of the present study is summarized in Fig. 1.

**Fig. 2:**
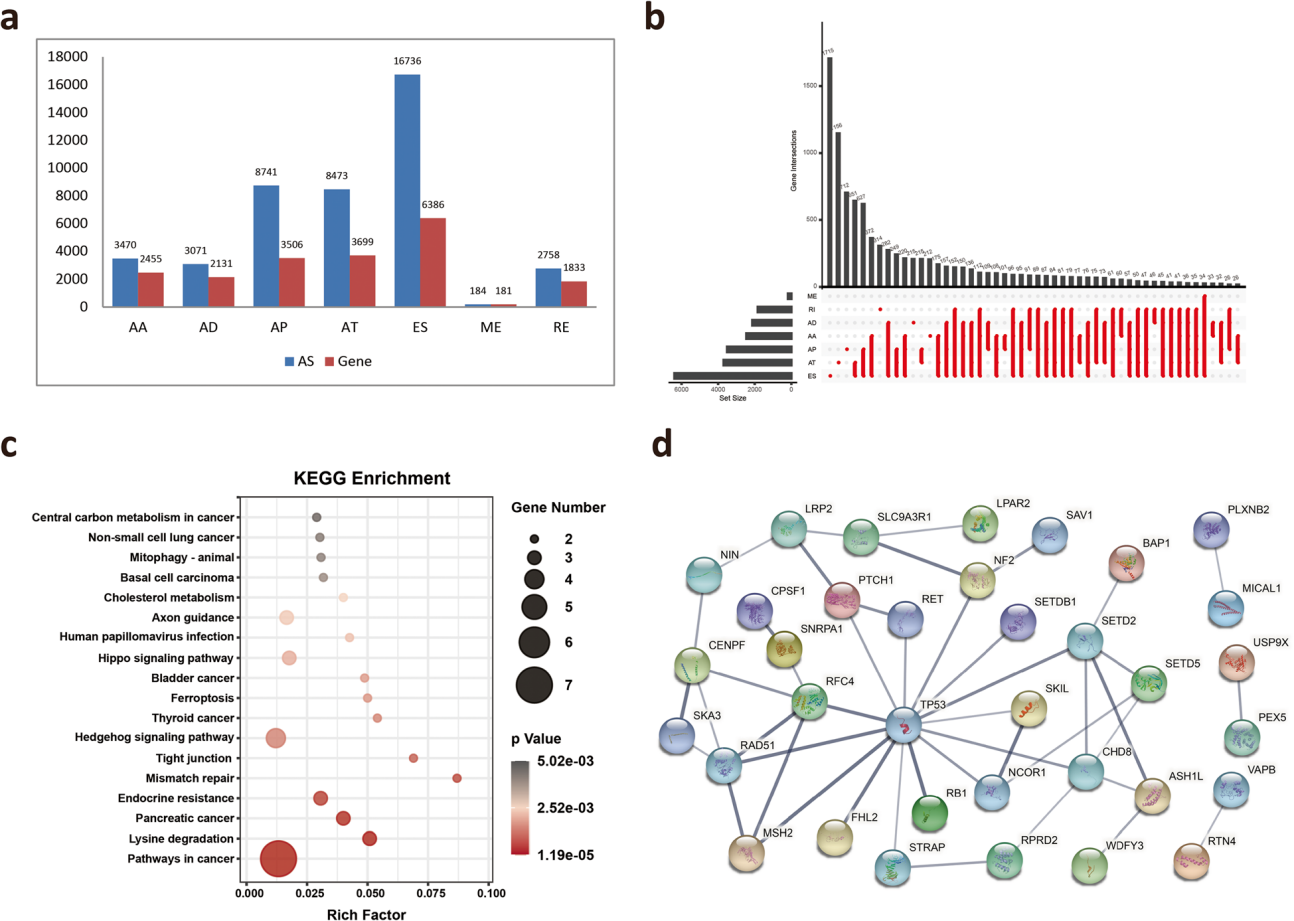
Overview of AS profiling and MM-specific exon skipped genes. **A** Number of AS events and parent genes for each AS event type in 83 MM patients. **B** UpSet plot of interactions among the seven types of AS events. **C** KEGG pathway analysis of 103 mutation-associated ES events from 80 genes in MM. The dot size represents the number of enriched genes, and adjusted *p* values are indicated by the color scale on the side. **D** Interaction network of 80 ES genes in MM. AA, alternate acceptor; AD, alternate donor; AP, alternate promoter; AT, alternate terminator; ES, exon skip; ME, mutually exclusive exon; RI, retained intron

A total of 87 MM patients from TCGA database were included in our study. The detailed clinical features of the patients are summarized in **Table S1**. Four patients with less than 30 days of follow-up were excluded. Integrated AS events were comprehensively analyzed based on their PSI values. We detected 43,433 AS events in 10,145 parent genes **(**Fig. 2**A)**. Because AS events influence gene translation and protein diversity, we selected these AS events to analyze the distributions of the genes involved in these events. These interactive sets were visualized by the UpSet plot. According to the splicing patterns, these AS events were divided into seven types. Among the seven patterns of alternative splicing, exon skipping (ES) events were the most frequent type **(**Fig. 2**B)**.

Accumulated evidence has reported that ES is the most common AS type and is associated with mutations leading to mesothelioma [31]. We next screened the ES-induced mutations from the ExonSkipDB database. Among these ES events, we obtained 103 mutation-associated ES events from 80 genes in MM **(****Table S2****)**. KEGG analysis of 80 genes revealed that “Pathway in cancer”, “Hedgehog signaling pathway” and “Hippo signaling pathway” were the most significant pathways **(**Fig. 2**C)**. To observe the relationship between these 80 genes, we mapped these genes to the STRING database to obtain the interaction of these genes using a score greater than 0.5 **(**Fig. 2**D)**. Among these mutated genes, only *BAP1, SETD2, SETDB1, NF2* and *TP53* have been reported in MM [16]. These ES events associated with gene mutations may play a critical role during MM development.

### Identification of survival-related AS events

**Fig. 3:**
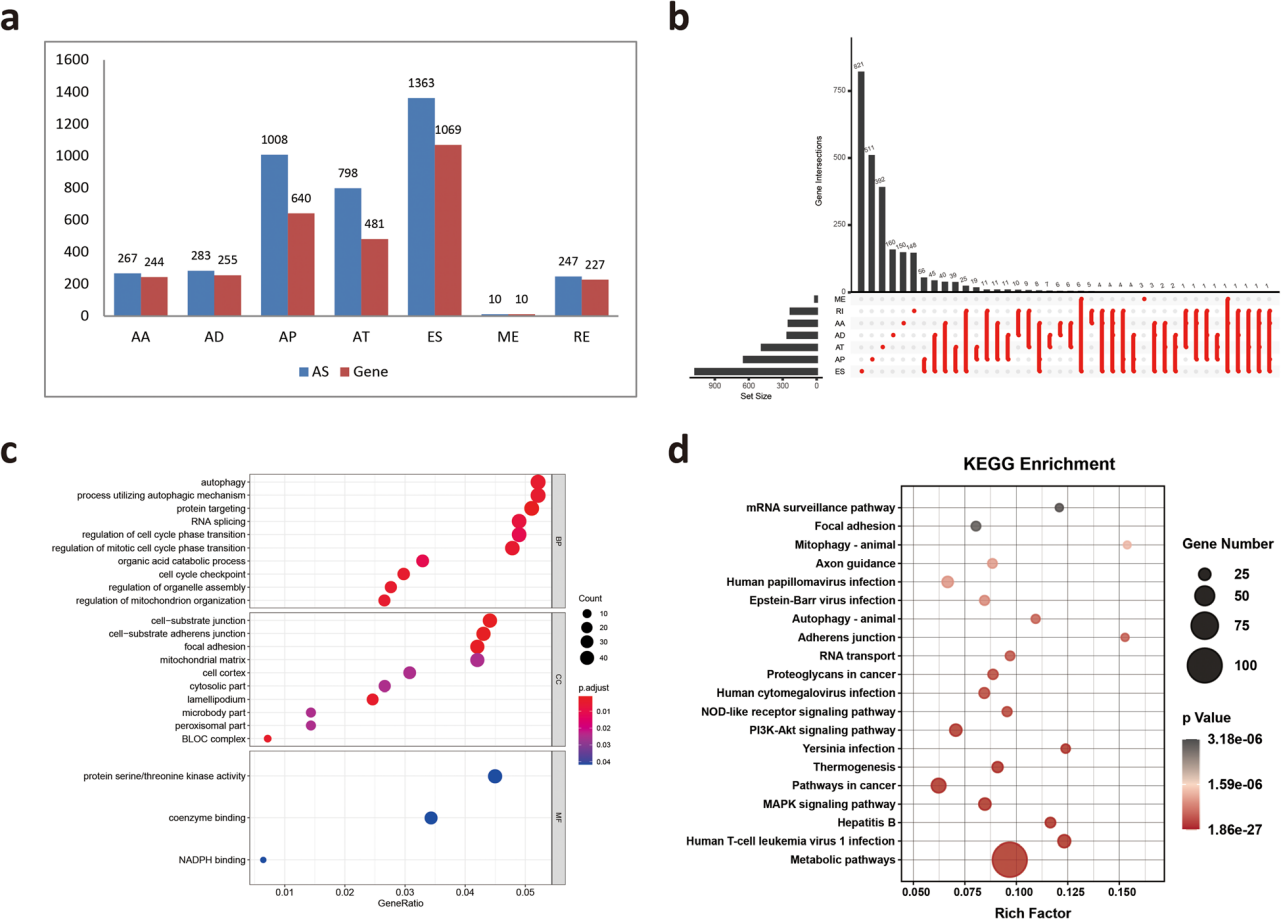
Overview of survival-related AS events and functional enrichment analyses of survival-related ES events genes. **A** Number of survival-related AS events and parent genes for each AS type. **B** UpSet plot showing the interactions among the seven types of survival-related AS events. **C** GO analysis of parental genes with survival-associated ES events. **D** KEGG pathway analysis of parental genes with survival-associated ES events

To investigate the relationship between AS and prognosis, univariate survival analysis between AS events and the prognosis of MM patients was performed on 43,433 AS events to identify survival-related AS events. A total of 3976 AS events were identified as candidate survival-related AS events, involving 2532 parent genes **(**Fig. 3**A)**. The distribution of these seven types of AS events and their intersections were quantitatively analyzed. The top 10 most significant survival-related AS events are shown using bubble plots in **Fig. S1**.

As expected, ES events accounted for the greatest proportion among the survival-related AS events **(**Fig. 3**B)**, suggesting that ES events are significantly correlated with prognosis. In addition, we focused on ES events because they resulted in loss of functional domains or frame shifting of the open reading frame (ORF), leading to a variety of human cancers. Our results identified 1363 survival-related ES events in 1069 ES-related genes. To explore the gene functions in ES events, the parent genes of the survival-related ES events were subjected to GO and KEGG enrichment analyses. KEGG analysis revealed that the enrichment of these genes was related to multiple pathways, including “Metabolic pathways”, “MAPK signaling pathway” and “PI3K-Akt signaling pathway” **(**Fig. 3**C)**. The top enriched GO terms for biological processes were “Autophagy”, “Process utilizing autophagic mechanism” and “RNA splicing”, indicating the active aberrant splicing patterns of MM. In addition, “Cell-substrate junction” and “Protein serine/threonine kinase activity” were also significantly enriched in cellular component and molecular function **(**Fig. 3**D)**.

### Construction of prognostic AS signatures for each AS type

**Fig. 4:**
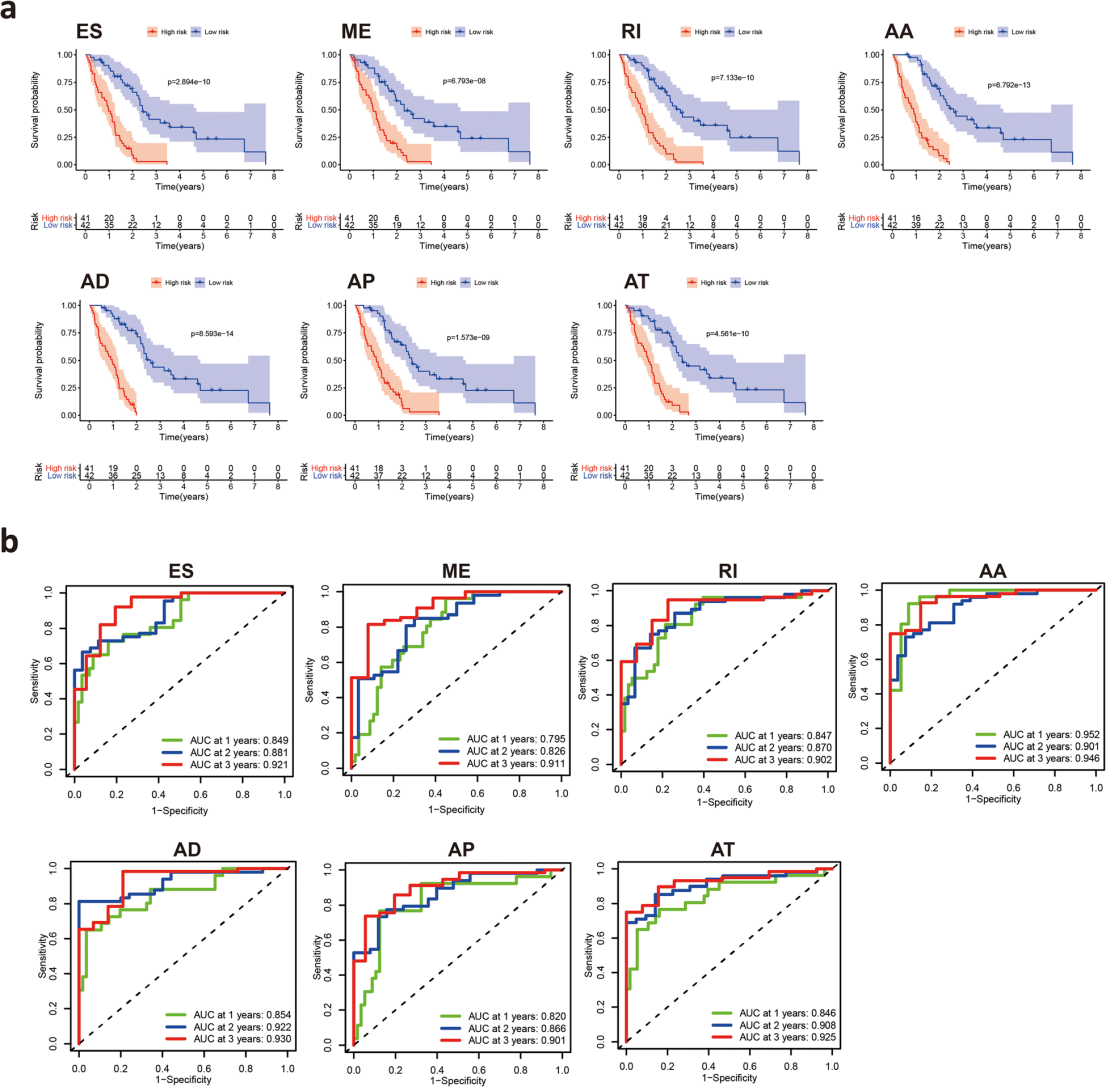
Kaplan-Meier plots and ROC curves of prognostic signatures for each AS type. **A** Kaplan-Meier survival analysis of OS between the low-risk group and the high-risk group in MM patients based on survival-related AS events. The red line indicates the high-risk group, whereas the blue line indicates the low-risk group. **B** Time-dependent ROC curves to evaluate the predictive performance of each prognostic signature at 3 years and 5 years

**Fig. 5:**
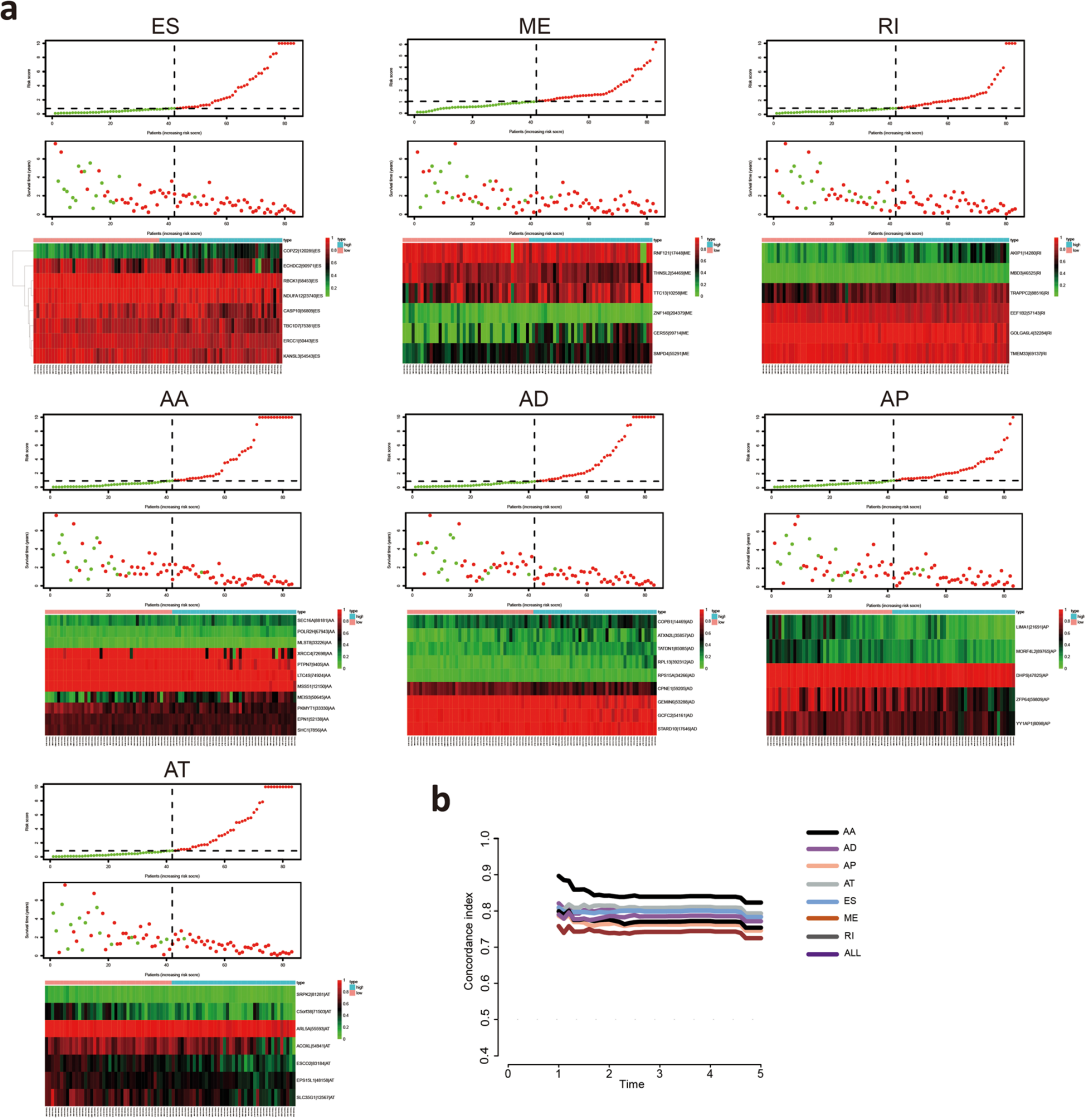
Assessment of the predictive ability of prognostic signatures. **A** MM patients were divided into the high-risk and low-risk groups based on the risk score. Distributions of the risk score, survival time and the expression heatmap of the candidate AS events of the seven significant prognostic signatures with AUCs > 0.9. **B** Calculation of the C-index of each AS prognostic signature

To examine the prognostic capacity of the survival-related AS events, LASSO regression was first performed to screen the most significant survival-related AS events from 3976 survival-related AS events. The results of the LASSO regression analyses are displayed in **Fig. S2**. Multivariate Cox regression modeling for independent prognostic factors was then performed to calculate a risk score for each patient, and detailed information on the prognostic signatures based on the seven types of AS events is shown in Table 1. According to the risk score, the MM patients were divided into low-risk and high-risk groups. Kaplan-Meier survival analysis demonstrated that patients in the low-risk group had a better prognosis than those in the high-risk group **(**Fig. 4**A)**. Time-dependent ROC analyses at 3 years and 5 years were conducted to validate the prognostic performance of the prognostic signatures. The AUC values of the ROC curve of all prognostic signatures were greater than 0.9, demonstrating excellent performance in prognosis prediction **(**Fig. 4**B)**. These seven prognostic signatures with AUCs ≥0.9 were selected for subsequent analysis. The information of the corresponding AS types of the candidate AS events as well as the survival time and living status ranked by the distribution of the risk score are displayed in Fig. 5**A**. In addition, the C-index of each AS prognostic signature was greater than 0.7 **(**Fig. 5**B).** The above results suggested that AS-based prognostic signatures with a risk score system may potentially be used as a novel method for classifying MM patients.

**Table 1:** Multivariate Cox analysis of prognostic AS signatures of each AS type

Gene symbol	Spliceseq ID	AS type	coef	HR	HR.95 L	HR.95H	pvalue
PKMYT1	33,330	AA	−4.62365	0.009817	0.000149	0.644696	0.030344
POLR2H	67,943	AA	12.70464	329,270.6	42.50452	2.55E+ 09	0.005425
EPN1	52,138	AA	−8.32915	0.000241	2.21E-08	2.630785	0.079083
MEIS3	50,645	AA	2.326581	10.24286	2.092053	50.1499	0.004095
SEC16A	88,181	AA	6.467639	643.9617	2.325998	178,283.4	0.024185
XRCC4	72,698	AA	−1.38736	0.249733	0.081034	0.769636	0.015695
LTC4S	74,924	AA	−43.1454	1.83E-19	1.65E-30	2.02E-08	0.000883
MSS51	12,150	AA	−17.2217	3.32E-08	2.55E-14	0.043206	0.016516
SHC1	7856	AA	−8.01876	0.000329	2.09E-08	5.189885	0.10394
PTPN7	9405	AA	−13.4042	1.51E-06	2.87E-09	0.000792	2.74E-05
MLST8	33,226	AA	−12.8429	2.64E-06	1.63E-11	0.42889	0.035881
RPL13	392,312	AD	9.429979	12,456.27	155.7049	996,491.4	2.47E-05
COPB1	14,469	AD	3.238635	25.49888	0.891659	729.1948	0.058366
TATDN1	85,085	AD	5.362926	213.3483	1.339859	33,971.85	0.038167
CPNE1	59,205	AD	−4.09642	0.016632	0.000334	0.827904	0.039909
GCFC2	54,161	AD	−10.1517	3.90E-05	5.93E-09	0.256718	0.02363
GEMIN6	53,288	AD	−10.9105	1.83E-05	8.47E-09	0.039375	0.005338
ATXN2L	35,857	AD	2.993779	19.96097	1.791751	222.3748	0.014927
RPS15A	34,266	AD	11.40439	89,714.7	0.932723	8.63E+ 09	0.051407
STARD10	17,646	AD	−24.3236	2.73E-11	2.27E-18	0.000329	0.003453
ECHDC2	90,971	ES	−1.24895	0.286807	0.05954	1.381561	0.119465
ERCC1	50,443	ES	−7.79417	0.000412	3.53E-07	0.480997	0.030535
COPZ2	120,285	ES	3.396763	29.86727	1.78974	498.4265	0.018017
RBCK1	58,453	ES	−17.8953	1.69E-08	7.55E-14	0.003788	0.004413
NDUFA12	23,740	ES	−3.30745	0.036609	0.000847	1.582308	0.085221
KANSL3	54,543	ES	−7.00065	0.000911	2.05E-05	0.04055	0.0003
CASP10	56,809	ES	−2.28213	0.102067	0.0061	1.707783	0.112368
TBC1D7	75,381	ES	−5.37593	0.004627	0.000122	0.175366	0.003748
ACOXL	54,941	AT	−2.76667	0.062871	0.013075	0.302304	0.000554
ESCO2	83,184	AT	−2.59645	0.074538	0.005148	1.079276	0.056908
ARL5A	55,593	AT	−31.2748	2.62E-14	3.68E-20	1.86E-08	5.37E-06
EPS15L1	48,158	AT	−5.22216	0.005396	0.000119	0.243627	0.007223
SRPK2	81,281	AT	−12.2981	4.56E-06	6.00E-11	0.346551	0.031971
C5orf38	71,503	AT	−3.80851	0.022181	0.003116	0.157876	0.000143
SLC35G1	12,567	AT	−2.08066	0.124848	0.009697	1.607419	0.110508
GOLGA6L4	32,284	RI	−6.81726	0.001095	1.56E-06	0.769218	0.041508
AKIP1	14,280	RI	2.88328	17.87281	1.894855	168.5813	0.011797
TRAPPC2	88,516	RI	3.320201	27.66591	1.281781	597.1397	0.034145
MBD3	46,525	RI	14.74354	2,529,512	305.7635	2.09E+ 10	0.001358
TMEM33	69,137	RI	−6.68218	0.001253	6.35E-07	2.471107	0.084302
EEF1B2	57,143	RI	−6.42918	0.001614	1.51E-06	1.729919	0.070917
CERS5	99,714	ME	1.045024	2.843466	1.051878	7.686538	0.039432
SMPD4	55,291	ME	2.387929	10.89091	0.826019	143.5948	0.069569
THNSL2	54,469	ME	−2.99269	0.050153	0.004231	0.594429	0.017678
ZNF140	204,379	ME	−5.46035	0.004252	4.34E-05	0.416614	0.019581
RNF121	17,448	ME	−1.70312	0.182114	0.050196	0.660716	0.00959
TTC13	10,258	ME	2.143182	8.526528	1.524471	47.68977	0.014687
ZFP64	59,809	AP	−1.49769	0.223647	0.040284	1.241632	0.086805
YY1AP1	8098	AP	−3.10861	0.044663	0.001998	0.998353	0.049879
DHPS	47,825	AP	−23.3753	7.05E-11	1.88E-19	0.026407	0.020299
LIMA1	21,691	AP	−2.42334	0.088625	0.008378	0.937537	0.044056
MORF4L2	89,765	AP	−3.76226	0.023231	0.000582	0.927201	0.045484

### Construction of a prognostic signature based on all AS types

**Fig. 6:**
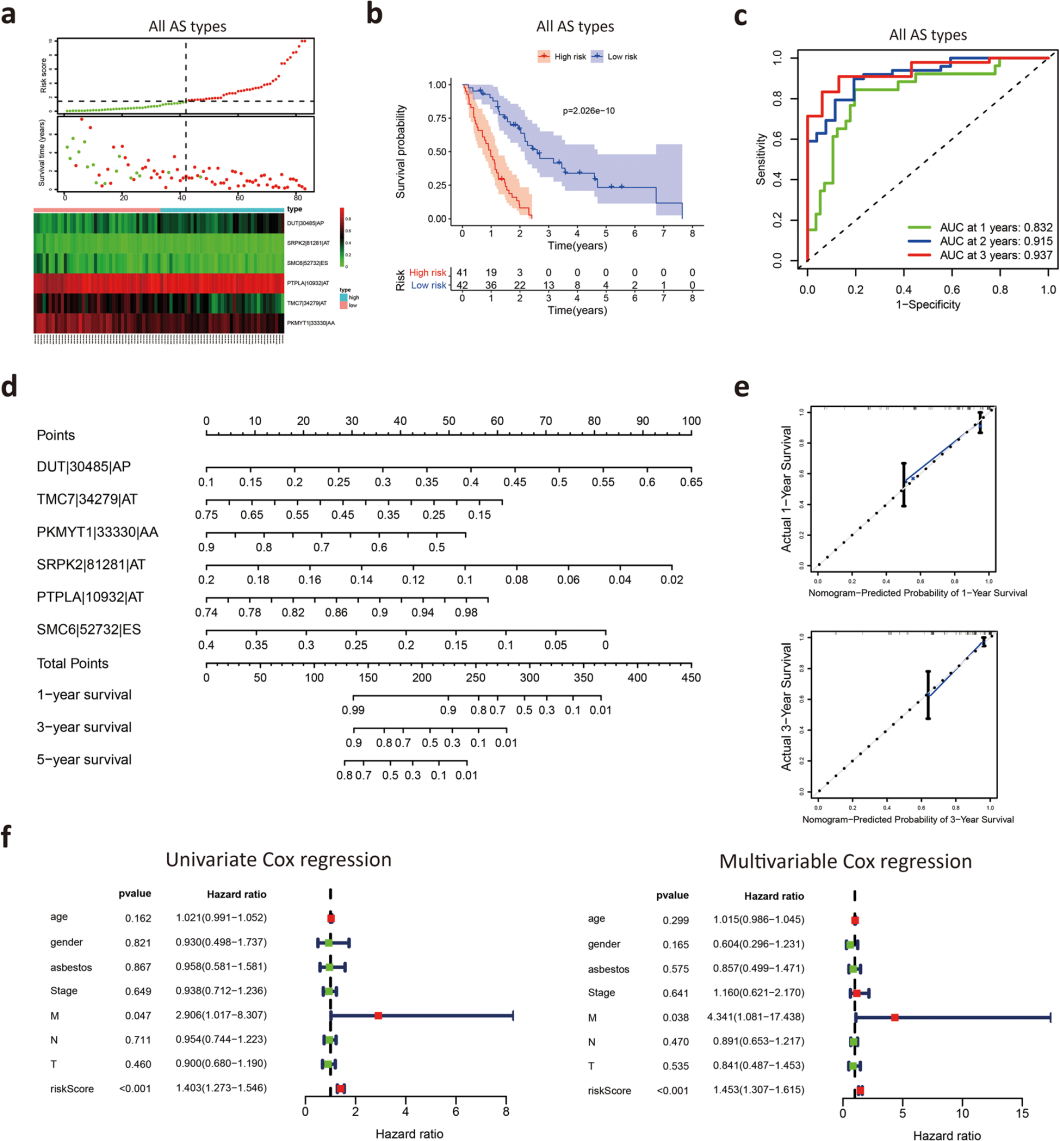
Construction of a prognostic model based on all AS events. **A** MM patients were divided into high-risk and low-risk groups according to the risk score model. **B** Kaplan-Meier survival analysis of MM patients in the low-risk and high-risk groups. **C** Time-dependent ROC curves to evaluate the predictive performance of the prognostic signature at 3 years and 5 years. **D** The nomogram was constructed based on AS events of the prognostic signature. **E** Calibration curves for predicting the three- and five-year survival probability of MM patients. **F** Univariate and multivariable Cox proportional hazards regression models were applied to evaluate the independence of the prognostic signature

Finally, the AS events were further limited to 6 key genes with AS events based on all types of AS events, and the details of the 6 AS events are shown in Table 2. We divided MM patients into high-risk and low-risk groups according to the median risk score (Fig. 6**A**). The Kaplan-Meier survival curve showed that patients with low risk had significantly longer survival times than those with high risk **(**Fig. 6**B**). The AUC values of the 6 AS event risk score models predicting the 3-year and 5-year survival rates reached 0.941 and 0.997, respectively **(**Fig. 6**C)**, representing high prediction accuracy. The 6 AS events were used to construct a nomogram based on Cox regression **(**Fig. 6**D)**, which predicted the 1-, 3- and 5-year survival status, supporting the nomogram suitability to predict the survival rate for MM patients. The calibration curves indicated that the nomogram had good prediction accuracy **(**Fig. 6**E)**.

**Table 2:** Multivariate Cox analysis of prognostic AS signatures based on all AS events

Gene symbol	Spliceseq ID	AS type	coef	HR	HR.95 L	HR.95H	pvalue
DUT	30,485	AP	2.54606589	12.7568182	0.85448400	190.449921	0.06489959
TMC7	34,280	AT	3.006121803	20.2088737	1.49090350	273.926902	0.02380556
COPB1	14,469	AD	2.982523553	19.7375626	0.95024497	409.969418	0.05398084
PKMYT1	33,330	AA	−3.82534411	0.02181092	0.00062199	0.76482814	0.03505836
TMCO3	26,380	ES	−9.32210286	8.94E-05	4.44E-08	0.18013857	0.01632685
PTPLA	10,932	AT	8.234517794	3768.82211	5.20565361	2,728,575.72	0.01424550
RASGRP3	53,188	AP	−1.74479491	0.17468080	0.05054900	0.60363971	0.00581895
PLEC	85,514	AP	−10.1222147	4.02E-05	3.09E-08	0.05232083	0.00567041
DUT	30,485	AP	2.54606589	12.7568182	0.85448400	190.449921	0.06489959

Next, we explored the correlation between the risk score and histologic subtypes. Patients with epithelial mesothelioma had a better prognosis than patients with other mesothelioma cell types **(****Fig. S3A****)**. We observed that the risk score was significantly decreased in patients with the epithelioid subtype **(****Fig. S3B****)**. We further analyzed the correlation between the risk score and clinical characteristics, including age, sex, T stage, N stage, M stage and clinical stage. There were no significant correlations between the risk score and these clinical characteristics **(****Fig. S3C****)**.

To verify the independent prognostic power of these signatures, clinicopathological parameters, including asbestos exposure, age, gender, T stage, N stage, M stage and clinical stage, were recorded as binary variables. Both univariable and multivariable Cox proportional hazards regression models were applied to evaluate the independent prognostic value of this prognostic model. The results from the univariate and multivariate Cox regression analyses demonstrated that the risk score significantly correlated with the survival of MM patients **(**Fig. 6**F)**. These results indicated that the 6 AS event-based prognostic signature had good accuracy in predicting the survival of patients with MM.

### Risk score and AS events are associated with immune infiltration level in the tumor microenvironment

**Fig. 7:**
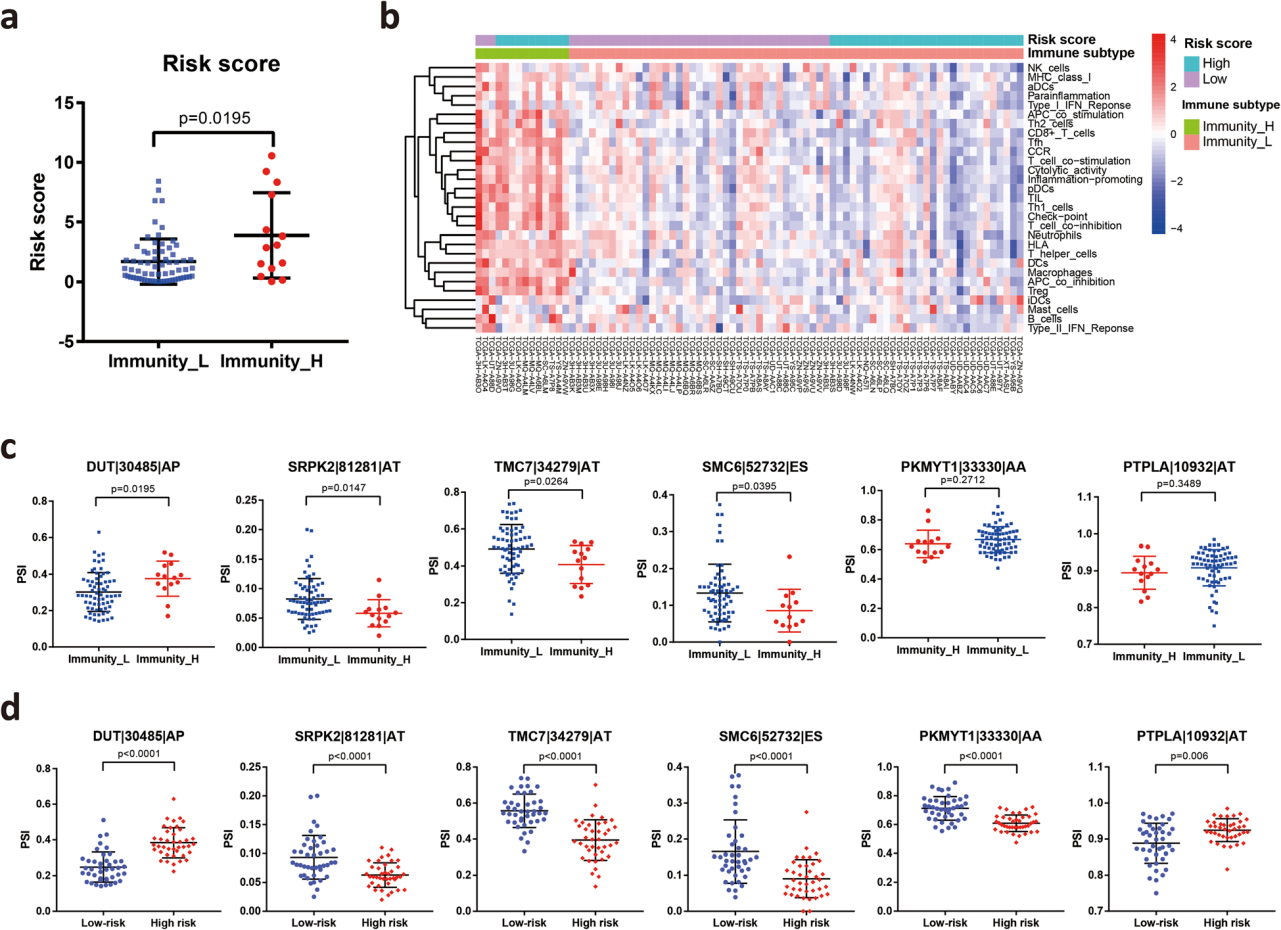
Relationship between risk scores and tumor-infiltrating immune cells in the tumor microenvironment. **A** Distribution of risk scores in the low- and high-immune infiltration subtypes. **B** Unsupervised clustering heat map showing the association between the risk score and the immune infiltration subtype of MM patients. **C** The PSI of 6 key AS events in two immune infiltration subtypes. **D** The PSI of 6 key AS events between the high-risk score group and the low-risk score group

Increasing evidence has indicated that AS alterations may affect immune cell infiltrations in the tumor microenvironment [32, 33]. To explore the association between the AS event-based risk score and the immune infiltration level of MM patients, we applied the ssGSEA score to cluster and classify the immunity status of MM patients. Patients were divided into high- and low-immune infiltration subtypes based on the immune score. Our analysis revealed that risk scores were higher in the high-immune infiltration subtype than in the low-immune infiltration subtype **(**Fig. 7**A)**. Unsupervised clustering clearly showed that most of the high immune infiltration patients belonged to the high-risk group **(**Fig. 7**B)**. Furthermore, the PSI of three AS events, namely, TMC7|34,279|AT, SRPK2|81,281|AT and SMC6|52,732|ES, increased in the low immune infiltration subtype, while the PSI of DUT|30,485|AP decreased in the low immune infiltration subtype **(**Fig. 7**C)**, which was consistent with the trend in the high-risk and low-risk groups **(**Fig. 7**D)**. We investigated the association between immune infiltration and histologic subtypes. Our results showed that biphasic mesothelioma patients displayed high immune infiltration compared to patients with epithelial mesothelioma **(Fig. S4A)**. These results demonstrated that the 6 AS event-based risk scores are negatively associated with the immune infiltration level of MM patients.

### Risk scores are correlated with immune cells in the tumor microenvironment

**Fig. 8:**
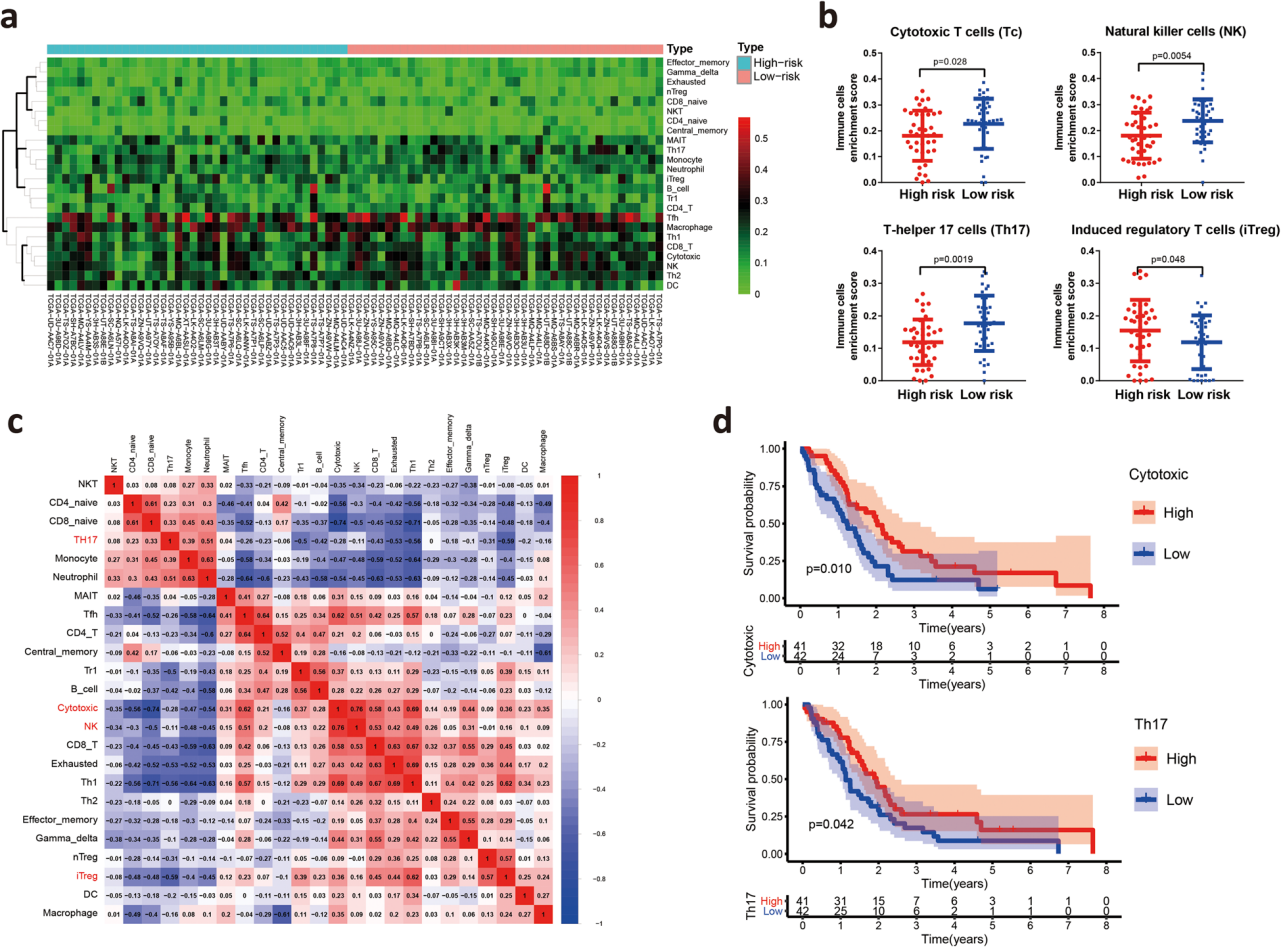
Risk scores are correlated with immune cells in the tumor microenvironment. **A** Estimation of the relative abundances of the 24 tumor-infiltrating immune cells by ImmuCellAI. **B** Comparisons of the abundances of cytotoxic T (Tc) cells, natural killer (NK) cells, T helper 17 (Th17) cells and induced regulatory T (iTreg) cells between the high-risk and low-risk groups. **C** Correlation analyses of 24 types of tumor-infiltrating immune cells in MM patients. **D** Survival analysis of patients with different infiltration of Th17 cells and cytotoxic T cells

Because the immune infiltration subtypes only reflected the overall proportion of immune infiltration and not the infiltration of specific immune cells, we further investigated the correlation between the risk score and the infiltration of 24 types of immune cells in the tumor microenvironment by ImmuCellAI **(**Fig. 8**A)**. Unexpectedly, differential analysis showed that the abundances of cytotoxic T (Tc) cells, natural killer (NK) cells and T-helper 17 (Th17) cells were higher in the low-risk group, whereas the abundances of induced regulatory T (iTreg) cells were decreased in the low-risk group **(**Fig. 8**B and Fig. S4B)**. Survival analysis found that high infiltration of both Th17 cells and cytotoxic T cells was associated with good prognosis, which was consistent with the above results that the low-risk group had better overall survival **(**Fig. 8**C)**. Correlation analyses of immune cells indicated that the numbers of NK cells and cytotoxic T cells exhibited strong positive correlations, while the numbers of Th17 cells were negatively correlated with the numbers of iTreg cells **(**Fig. 8**D)**. In addition, Th17 cells were enriched in epithelial mesothelioma compared to biphasic mesothelioma **(Fig. S4C)**. Together, the above results revealed that a low-risk score is correlated with the infiltration of immune cells, especially effector T cells, in MM patients.

### Survival-related splicing regulatory network

**Fig. 9:**
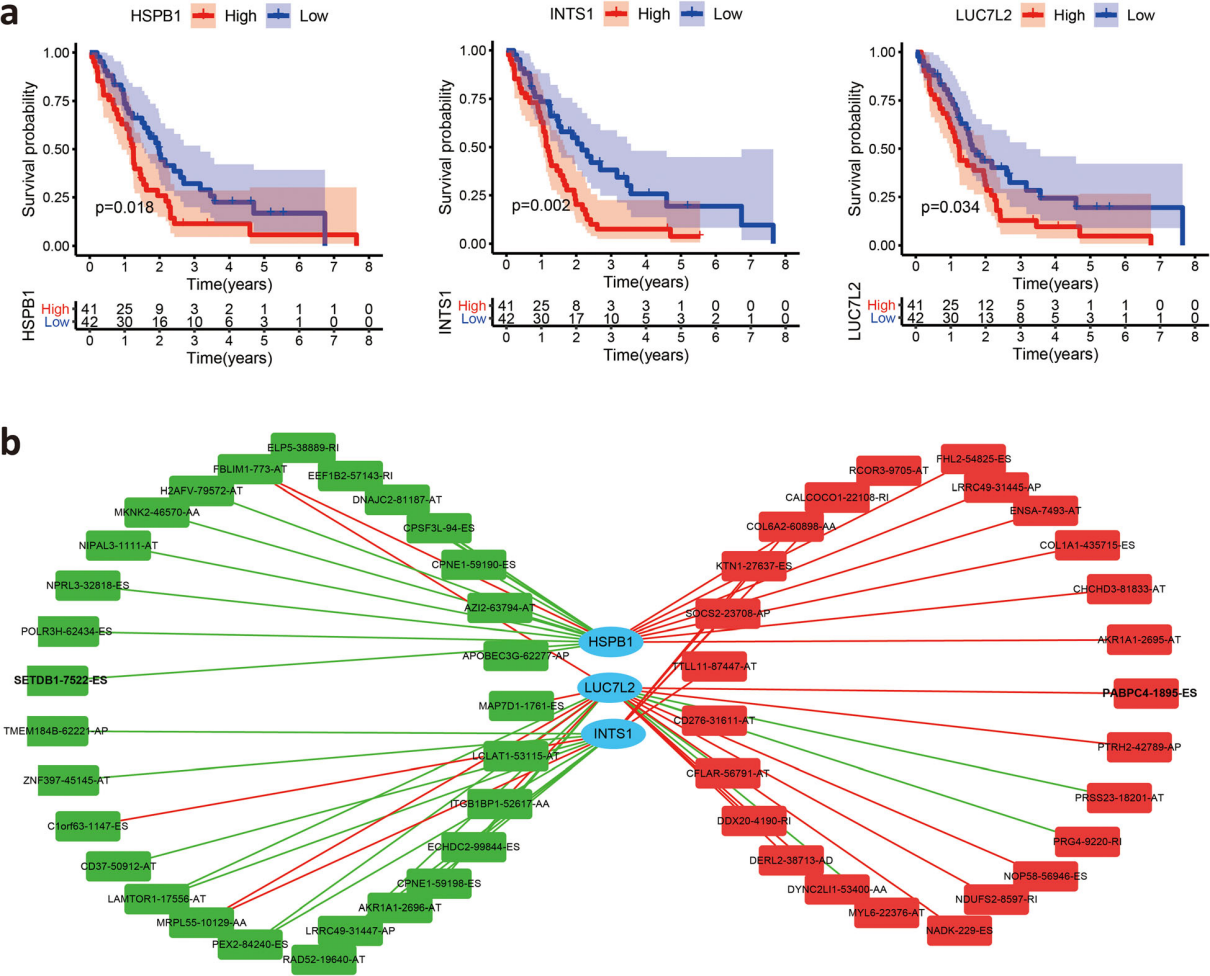
Prognostic SFs and the splicing regulation network. **A** Kaplan-Meier survival curves of significant prognosis-related SFs, including *HSPB1, INTS1* and *LUC7L2*. **B)** Construction of the interaction network of survival-related SFs and survival-related AS events. The positive/negative correlations between the expression of SFs and PSI values for AS events are represented with red/green lines

Splicing factors are one of the vital regulatory factors of AS events. A list of 119 SFs was obtained from a previous study [30]. Univariate Cox regression analyses of these 119 SFs based on TCGA data showed that 13 out of 119 SFs were associated with OS in MM patients (Table 3). Survival analyses suggested that 3 out of 13 survival-associated SFs possessed the ability to predict the survival of patients with MM, including *HSPB1, INTS1* and *LUC7L2* **(**Fig. 9**A)**. Correlation analysis was performed to evaluate the correlation between survival-associated SF genes and prognosis-related AS events in each splicing type. In total, 29 downregulated AS events (green rectangles) and 26 upregulated AS events (yellow rectangles) were correlated with the 3 survival-associated SFs in this network **(**Fig. 9**B)**. Among these 55 prognosis-related AS events, 3 ES events (FHL2–54825-ES, PABPC4–1895-ES and SETDB1–7522-ES) overlapped with 103 mutation-associated ES events in MM **(Fig. S5)**. Considering the above results, MM-related genes and AS events play vital roles in MM biology, but further research is needed. The construction of a correlation network provided evidence for deciphering the regulatory mechanisms of AS events in MM.

**Table 3:** Survival- associated splicing factors from univariate Cox regression analysis

Splicing factor	HR	HR.95 L	HR.95H	pvalue
CSN3	1.026871	1.009237	1.044813	0.002697
U2AF2	1.047588	1.010588	1.085943	0.011276
INTS1	1.036617	1.007235	1.066855	0.01423
PRPF8	1.039691	1.006003	1.074507	0.020554
RBM3	1.013026	1.001595	1.024588	0.025405
SRRT	1.053552	1.005442	1.103963	0.028702
CWC25	1.155498	1.014415	1.316202	0.029601
EFTUD2	1.061764	1.00437	1.122438	0.034537
LUC7L2	1.17085	1.005572	1.363294	0.042201
HSPB1	1.680284	1.207518	2.338145	0.00208
SPEN	1.112426	1.002723	1.234132	0.044294
ZNF131	1.393721	1.002499	1.937615	0.048289
RBM7	0.794598	0.631783	0.999372	0.049376

## Discussion

Changes in AS events are frequently observed in cancer and are beginning to be recognized as critical signatures of tumor development, differentiation and treatment [14]. MM is an aggressive tumor with high chemoresistance and poor survival [34]. Due to the low incidence of MM, there has been little progress in the knowledge of the molecular mechanisms associated with MM. In the present study, we provided a systematic landscape of AS events in MM. Based on survival-related AS events, we constructed prognostic AS signatures that stratified MM patients into low-risk and high-risk groups with distinct survival outcomes. Moreover, we investigated the association among risk score, histologic subtype and immune system, providing further insights into successful establishment of diagnostic, therapeutic and prognostic systems.

The aberrant splicing of pre-mRNA in tumor cells contributes to multiple cell functions, such as proliferation, invasion, metastasis and drug resistance, representing potential tumor-specific biological markers for clinical application [35, 36]. Tumor cells have cancer type-specific and subtype-specific alterations during the process of splicing, which have prognostic value and contribute to every hallmark of cancer progression [37]. Kahles and colleagues analyzed more than 8000 tumors across 32 cancer types and found thousands of alternative splicing events not detectable in nonmalignant tissues, which likely lead to cancer-specific markers and neoantigens [38]. Recent studies have focused on investigating the survival prognostic value of AS in cancers. Numerous studies have conducted SpliceSeq analyses to generate AS profiles for cancer prognosis monitoring with scores, including papillary thyroid carcinoma [39], colorectal cancer [40], non-small cell lung cancer [41], melanoma [42], hepatocellular carcinoma [43] and kidney cancer [44]. Our study added to the comprehensive understanding of patients with MM and identified survival-related AS signatures using high-throughput data. We systematically examined the prognostic value of AS events in MM patients. A total of 3976 AS events were identified as candidate survival-related AS events, and 2532 parent genes were involved. As mentioned above, the lost protein features of ES events are important for evaluating the functional effects of MM-related genes on tumorigenesis. Among the survival-related ES genes, *NF2, BAP1, PEX5, RAD51, FHL2, XPO6, RHOT1, MEGF6, PLXNB2, SETD5* and *WIZ* overlapped with MM-specific ES genes, which may play a critical role during MM development. Several previous genomic studies have reported that gene fusions and splice alterations are recurrent mechanisms leading to inactivation of *NF2* and *BAP1* in MM, and the present study provided additional data interpretation to the results in these previous studies [16, 45]. Moreover, we identified several potential biological pathways associated with survival-related ES genes, such as the MAPK signaling pathway, which has been found to be the most commonly affected biological pathway in MM patients [16] and in animal models of MM [46].

The relevance of cancer-specific AS events for serving as prognostic biomarkers and therapeutic targets is gaining recognition [16]. During the last decade, tremendous efforts have been devoted to integrating genome-wide prognostic biomarkers to improve the prognosis and diagnosis of MM [47, 48]. Hmeljak et al. conducted a comprehensive integrated genomic study of malignant pleural mesothelioma, showing higher aurora kinase mRNA expression in the poor prognosis subset [45]. Raphael et al. identified alterations in the Hippo, mTOR, histone methylation, RNA helicase and p53 signaling pathways in malignant pleural mesothelioma [16]. However, screening studies related to survival based on the selection of AS events and the establishment of prognostic models have not been widely performed in MM. Importantly, the present study constructed prognostic signatures based on AS events for monitoring the prognosis of MM patients. Kaplan-Meier analysis showed that the difference in OS between the low-risk and high-risk patients stratified according to the risk score was remarkable. Furthermore, time-dependent ROC curves demonstrated robust and excellent performance. Our results suggested that AS events have great potential significance in predicting the prognosis of MM patients.

Studies have reported that aberrant AS events are involved in a variety of tumor processes, including immune destruction [49, 50]. Neoepitopes derived from aberrant AS events are recognized by T lymphocytes to induce antitumor immune responses [51]. Thus, we analyzed the association between the risk score system based on 6 AS events and the immune infiltration level in the immune microenvironment. Although not all 6 AS events had the same trend, both risk score and 4 in 6 sample events were associated with the immune infiltration level. We discovered the correlation between the risk score system and the immune infiltration level in the immune microenvironment and showed that a low-risk score was significantly associated with upregulated cytotoxic T (Tc) cells, natural killer (NK) cells and T-helper 17 (Th17) cells. These effector T cells may be activated by neoepitopes derived from aberrant AS events, which may partially explain the correlation between the low-risk score and high infiltration of effector T cells in MM patients. Among the 6 key AS genes, the *SMC6* gene has been reported to be involved in DNA repair and checkpoint responses [52]. The *SRPK2* gene has been reported to be oncogenic, promoting the growth, migration and tumorigenicity of several malignancies [53, 54]. Aberrant AS of genes, such as *SRPK2* and *SMC6,* might be related to the production of new antigens and the induction of immune system activation in low-risk patients. The results indicated that the infiltration of immune cells altered by 6 key AS events provided a potential indicator to assess the immune stage of MM patients and predict the effect of immunotherapy.

Splicing factors are one of the crucial regulatory factors of AS events as they affect the binding of exon selection and splicing sites [55]. The spliceosome, which consists of five small nuclear RNAs, is the location of AS [56]. Alterations in spliceosomal components that influence splicing have been described in a variety of cancers [57]. Altered SFs in MM are considered independent molecules involved in carcinogenesis [16, 58]. Previous studies have reported that SF3B1 mutations affect the splicing of *p. Lys700Glu* in a mesothelioma cell line [59]. Raphael et al. identified mesothelioma tumors with missense mutations in *SF3B1*, which encodes a splice factor that is involved in branch-point recognition and *U2-snRNP* assembly [16]. In the present study, we identified three SFs that were significantly associated with MM patient survival, including *HSPB1, INTS1* and *LUC7L2*. Splicing correlation network analysis revealed interactive regulated nodes, suggesting the important positions of these SFs in the SF-AS network. The *HSPB1* gene is a member of the heat shock protein family and is strongly associated with the growth and survival of MM [60, 61]. A previous study has demonstrated that there is a directed protein interaction between *HSPB1* and *SETDB1* [62]. *SETDB1* is a survival ES gene that has been reported to be significantly mutated in MM. LUC7L2 is a spliceosomal protein that interacts with U1 snRNP to recognize 5′ splice sites [63]. However, the function of LUC7L2 is not well characterized, and the majority of the protein is based on its ortholog splicing factor, LUC7, which is involved in the recruitment and interaction of SF. The BioGRID (https://thebiogrid.org/) database indicated that there is a physical interaction between *LUC7L2* and *PABPC4*. Further studies are needed to explore the specificity and mechanisms of the *HSPB1* gene in MM.

Although our study identified several AS events that theoretically impact the prognosis of MM, our study still has some limitations. The present study was based on bioinformatics methods, and the results were not confirmed by experiments. In addition, the sample size in our study was limited, and further internal and external validations of the prognostic model are necessary.

## Conclusions

In summary, our study established prognostic signatures based on survival-related AS events, and prognostic signatures based on key AS events may serve as an effective risk model to predict the survival of MM patients. Furthermore, we investigated the correlation among risk score, histologic subtype and immune landscape. These results represented a novel direction for immunotherapeutic research and provided potential targets for personalized therapeutic intervention. In addition, the further identification of prognostic SFs and construction of the SF-AS network will pave the way for further investigation of the splicing-related mechanisms in MM.

## Supplementary Information

**Additional file 1: Table S1.** Clinicopathological characteristics of 83 MM patients from TCGA database. **Table S2** 103 mutation-associated ES events from 80 genes in MM. **Fig. S1**: Bubble plots of the top 10 significant survival-related AS events. **Fig. S2:** LASSO regression analysis was used to filter the modeling AS events from 3976 survival-related AS events. **Fig. S3: A** Kaplan-Meier survival analysis of OS between epithelioid and biphasic subtype in MM patients. **B** (Left) The distribution of risk scores between epithelioid and biphasic subtypes. (Right) The distribution of histologic subtypes between low-risk and high-risk groups. **C** The violin plot of correlation between risk score and clinical characteristics including clinical stage, gender, age, T stage, N stage and M stage. **Fig. S4: A** The distribution of immune infiltration level between epithelioid and biphasic subtypes. **B** Comparisons of the abundances of 24 types of tumor-infiltrating immune cells between low-risk and high-risk groups. **C** Comparisons of the abundances of 24 types of tumor-infiltrating immune cells between epithelioid and biphasic subtypes. **Fig. S5:** The Venn plot to identify the overlapped genes related to parent genes of 55 prognosis-related AS events and 103 mutation-associated ES events in MM.

## Data Availability

All data obtained for this study can be found in TCGA (https://portal.gdc.cancer.gov/). The percent spliced in (PSI) values of AS events were downloaded from TCGA SpliceSeq (https://bioinformatics.mdanderson.org/TCGASpliceSeq).

## Abbreviations

AS: Alternative splicing
MM: Malignant mesothelioma
ROC: Receiver operating characteristic
SF: Splicing factors
AUC: Area under the curve
OS: overall survival
PSI: Percent spliced in
AD: Alternate Donor site
AA: Alternate Acceptor site
AT: Alternate Terminator
AP: Alternate Promoter
ME: Mutually Exclusive Exons
ES: Exon Skip
RI: Retained Intron
GO: Gene ontology
KEGG: Kyoto Encyclopedia of Genes and Genomes
LASSO: Least absolute shrinkage and selection operator
ssGSEA: single-sample gene-set enrichment analysis
ImmuCellAI: Immune Cell Abundance Identifier
