# Supplementary material for: Systemic characterization of alternative splicing related to prognosis and immune infiltration in malignant mesothelioma
Source: BMC Cancer. 2021 Jul 22;21:848. doi: 10.1186/s12885-021-08548-3 (PMC8299698; doi:10.1186/s12885-021-08548-3)
Supplement: Supplementary file 1 — Additional file 1: Table S1. Clinicopathological characteristics of 83 MM patients from TCGA database. Table S2 103 mutation-associated ES events from 80 genes in MM. Fig. S1: Bubble plots of the top 10 significant survival-related AS events. Fig. S2: LASSO regression analysis was used to filter the modeling AS events from 3976 survival-related AS events. Fig. S3: A Kaplan-Meier survival analysis of OS between epithelioid and biphasic subtype in MM patients. B (Left) The distribution of risk scores between epithelioid and biphasic subtypes. (Right) The distribution of histologic subtypes between low-risk and high-risk groups. C The violin plot of correlation between risk score and clinical characteristics including clinical stage, gender, age, T stage, N stage and M stage. Fig. S4: A The distribution of immune infiltration level between epithelioid and biphasic subtypes. B Comparisons of the abundances of 24 types of tumor-infiltrating immune cells between low-risk and high-risk groups. C Comparisons of the abundances of 24 types of tumor-infiltrating immune cells between epithelioid and biphasic subtypes. Fig. S5: The Venn plot to identify the overlapped genes related to parent genes of 55 prognosis-related AS events and 103 mutation-associated ES events in MM. [file 12885_2021_8548_MOESM1_ESM.docx]

Table S1 Clinicopathological characteristics of 83 MM patients from TCGA database.

| Clinicopathological characteristics | Value |
| --- | --- |
| Gender, n(%) |  |
| Female | 14 (18.4%) |
| Male | 69 (81.6%) |
| Histologic subtypes, n(%) |  |
| Epithelioid | 58 (69.9%) |
| Sarcomatoid | 0 (0%) |
| Biphasic | 21 (25.3%) |
| Unknown | 4 (4.8%) |
| TCGA stage, n(%) |  |
| Stage I | 9 (11.5%) |
| Stage II | 15 (18.4%) |
| Stage III | 44 (51.7%) |
| Stage IV | 15 (18.4%) |
| T stage, n(%) |  |
| T1 | 13 (16.1%) |
| T2 | 25 (29.9%) |
| T3 | 31 (36.8%) |
| T4 | 12 (14.9%) |
| Unknown | 1 (2.3%) |
| N stage, n(%) |  |
| N0 | 43 (50.6%) |
| N1 | 9 (11.5%) |
| N2 | 25 (29.9%) |
| N3 | 3 (3.45%) |
| Nx | 3 (4.6%) |
| M stage, n(%) |  |
| M0 | 55 (65.5%) |
| M1 | 3 (3.5%) |
| Mx | 25 (31%) |
| Survival, n(%) |  |
| Alive | 16 (19.5%) |
| Dead | 67 (80.5%) |
| Overall survival (Days ± SD) | 645 ± 543 |

**Table S2** 103 mutation-associated ES events from 80 genes in MM.

| Gene | Skipped exon's start | Skipped exon's end | Mutation type | Gene | Skipped exon's start | Skipped exon's end | Mutation type |
| --- | --- | --- | --- | --- | --- | --- | --- |
| TP53 | 7576853 | 7576926 | Nonsense_Mutation | SLC44A4 | 31842498 | 31842623 | Frame_Shift_Del |
| RANBP10 | 67805937 | 67805989 | Nonsense_Mutation | SNRPA1 | 101825172 | 101825265 | Frame_Shift_Ins |
| SLC25A19 | 73274233 | 73274416 | Nonsense_Mutation | GAS2L1 | 29704077 | 29704728 | Frame_Shift_Del |
| RANBP10 | 67805937 | 67805989 | Nonsense_Mutation | GAS2L1 | 29704049 | 29704728 | Frame_Shift_Del |
| DTWD1 | 49917310 | 49917628 | Nonsense_Mutation | RHOT1 | 30529789 | 30529919 | Frame_Shift_Del |
| SAV1 | 51131897 | 51132337 | Nonsense_Mutation | MICAL1 | 109770860 | 109770986 | Frame_Shift_Del |
| PEX5 | 7351607 | 7351709 | Nonsense_Mutation | STRAP | 16048293 | 16048430 | Frame_Shift_Del |
| BAP1 | 52439781 | 52439928 | Nonsense_Mutation | CCDC134 | 42209268 | 42209449 | Frame_Shift_Del |
| NIN | 51219236 | 51219451 | Nonsense_Mutation | KLHL25 | 86311248 | 86313051 | Frame_Shift_Del |
| RAD51 | 41021703 | 41021832 | Nonsense_Mutation | MEGF6 | 3428114 | 3428251 | Frame_Shift_Ins |
| SKA3 | 21729832 | 21729950 | Nonsense_Mutation | BAP1 | 52441190 | 52441332 | Frame_Shift_Del |
| NCOR1 | 16062074 | 16062187 | Nonsense_Mutation | PLXNB2 | 50727946 | 50729026 | Frame_Shift_Ins |
| CPSF1 | 145621813 | 145621956 | Nonsense_Mutation | SETD2 | 47129603 | 47129737 | Frame_Shift_Ins |
| SPTAN1 | 131390204 | 131390221 | Nonsense_Mutation | RB1 | 48936951 | 48937093 | Frame_Shift_Del |
| SLC26A10 | 58016570 | 58016717 | Nonsense_Mutation | BAP1 | 52436795 | 52436956 | Frame_Shift_Del |
| NF2 | 30067815 | 30067937 | Nonsense_Mutation | BAP1 | 52436795 | 52436887 | Frame_Shift_Del |
| FHL2 | 105984027 | 105984196 | Nonsense_Mutation | VAPB | 57015963 | 57016139 | Frame_Shift_Ins |
| IL11RA | 34656736 | 34656905 | Nonsense_Mutation | SETD5 | 9475529 | 9475634 | Frame_Shift_Ins |
| SLC3A2 | 62652649 | 62652829 | Nonsense_Mutation | CACNA2D | 81693620 | 81693670 | Frame_Shift_Del |
| SLC3A2 | 62652649 | 62653080 | Nonsense_Mutation | DPY19L3 | 32945845 | 32945946 | Frame_Shift_Del |
| RANBP10 | 67805937 | 67805989 | Nonsense_Mutation | USP9X | 41022043 | 41022130 | Frame_Shift_Del |
| BAP1 | 52439781 | 52439928 | Nonsense_Mutation | NMNAT2 | 183253845 | 183253925 | Frame_Shift_Ins |
| C9orf85 | 74586421 | 74586534 | Nonsense_Mutation | SUPT20H | 37598172 | 37598330 | Frame_Shift_Del |
| WDFY3 | 85758082 | 85758243 | Nonsense_Mutation | SKIL | 170077487 | 170079217 | Frame_Shift_Del |
| LPAR2 | 19737352 | 19738093 | Nonsense_Mutation | BAP1 | 52436795 | 52436956 | Frame_Shift_Ins |
| LRRIQ1 | 85500300 | 85500393 | Nonsense_Mutation | BAP1 | 52436795 | 52436887 | Frame_Shift_Ins |
| SETD2 | 47098311 | 47098980 | Nonsense_Mutation | SLC26A8 | 35959436 | 35959585 | Frame_Shift_Del |
| RANBP10 | 67805937 | 67805989 | Nonsense_Mutation | SETD2 | 47125210 | 47125872 | Frame_Shift_Del |
| SETD2 | 47098311 | 47098980 | Nonsense_Mutation | BAP1 | 52437432 | 52437910 | Frame_Shift_Del |
| PTCH1 | 98238316 | 98238441 | Nonsense_Mutation | WDR53 | 196287867 | 196288362 | Frame_Shift_Del |
| XPO6 | 28123138 | 28123327 | Nonsense_Mutation | RFC4 | 186508115 | 186508195 | Frame_Shift_Ins |
| LRP2 | 170003249 | 170003469 | Nonsense_Mutation | LOXL3 | 74763836 | 74764055 | Frame_Shift_Ins |
| PPM1F | 22285520 | 22285663 | Nonsense_Mutation | SFTPB | 85892729 | 85892917 | Frame_Shift_Del |
| NEO1 | 73566152 | 73566346 | Nonsense_Mutation | BAP1 | 52439781 | 52439928 | Frame_Shift_Del |
| ASH1L | 155429588 | 155429689 | Nonsense_Mutation | RTN4 | 55252222 | 55254621 | Frame_Shift_Ins |
| BAP1 | 52437432 | 52437910 | Nonsense_Mutation | RET | 43597790 | 43598077 | Frame_Shift_Del |
| WDR35 | 20162056 | 20162088 | Nonsense_Mutation | TDO2 | 156825170 | 156825275 | Frame_Shift_Ins |
| MAP3K9 | 71202678 | 71202746 | Nonsense_Mutation | NF2 | 30038191 | 30038274 | Frame_Shift_Ins |
| NF2 | 30032740 | 30032865 | Nonsense_Mutation | POGLUT1 | 119190156 | 119190299 | Frame_Shift_Del |
| RPRD2 | 150418702 | 150418877 | Nonsense_Mutation | EPB41L1 | 34773039 | 34773257 | Frame_Shift_Del |
| FCRL3 | 157650771 | 157650889 | Nonsense_Mutation | RANBP3L | 36268295 | 36268369 | Frame_Shift_Del |
| ELF5 | 34515026 | 34515259 | Nonsense_Mutation | SERINC5 | 79473137 | 79473219 | Frame_Shift_Ins |
| MSH2 | 47637233 | 47637511 | Nonsense_Mutation | BAP1 | 52442490 | 52442622 | Frame_Shift_Del |
| DNAH6 | 84774616 | 84774736 | Nonsense_Mutation | EXTL1 | 26357951 | 26358057 | Frame_Shift_Del |
| IQGAP2 | 75993812 | 75993972 | Nonsense_Mutation | CENPF | 214817900 | 214820743 | Frame_Shift_Ins |
| SETDB1 | 150922934 | 150923566 | Nonsense_Mutation | THADA | 43459852 | 43460021 | Frame_Shift_Del |
| SETDB1 | 150922934 | 150923566 | Frame_Shift_Del | ATP13A3 | 194140608 | 194140696 | Frame_Shift_Ins |
| NF2 | 30035079 | 30035201 | Frame_Shift_Del | ST8SIA4 | 100222047 | 100222304 | Frame_Shift_Del |
| HDLBP | 242194789 | 242194926 | Frame_Shift_Ins | RELL2 | 141019487 | 141019862 | Frame_Shift_Del |
| HDLBP | 242194789 | 242194995 | Frame_Shift_Ins | WIZ | 15538921 | 15539319 | Frame_Shift_Del |
| CHD8 | 21898960 | 21900017 | Frame_Shift_Ins | MEGF8 | 42838159 | 42838365 | Frame_Shift_Del |
| SLC9A3R1 | 72763075 | 72763115 | Frame_Shift_Del |  |  |  |  |

Fig. S1


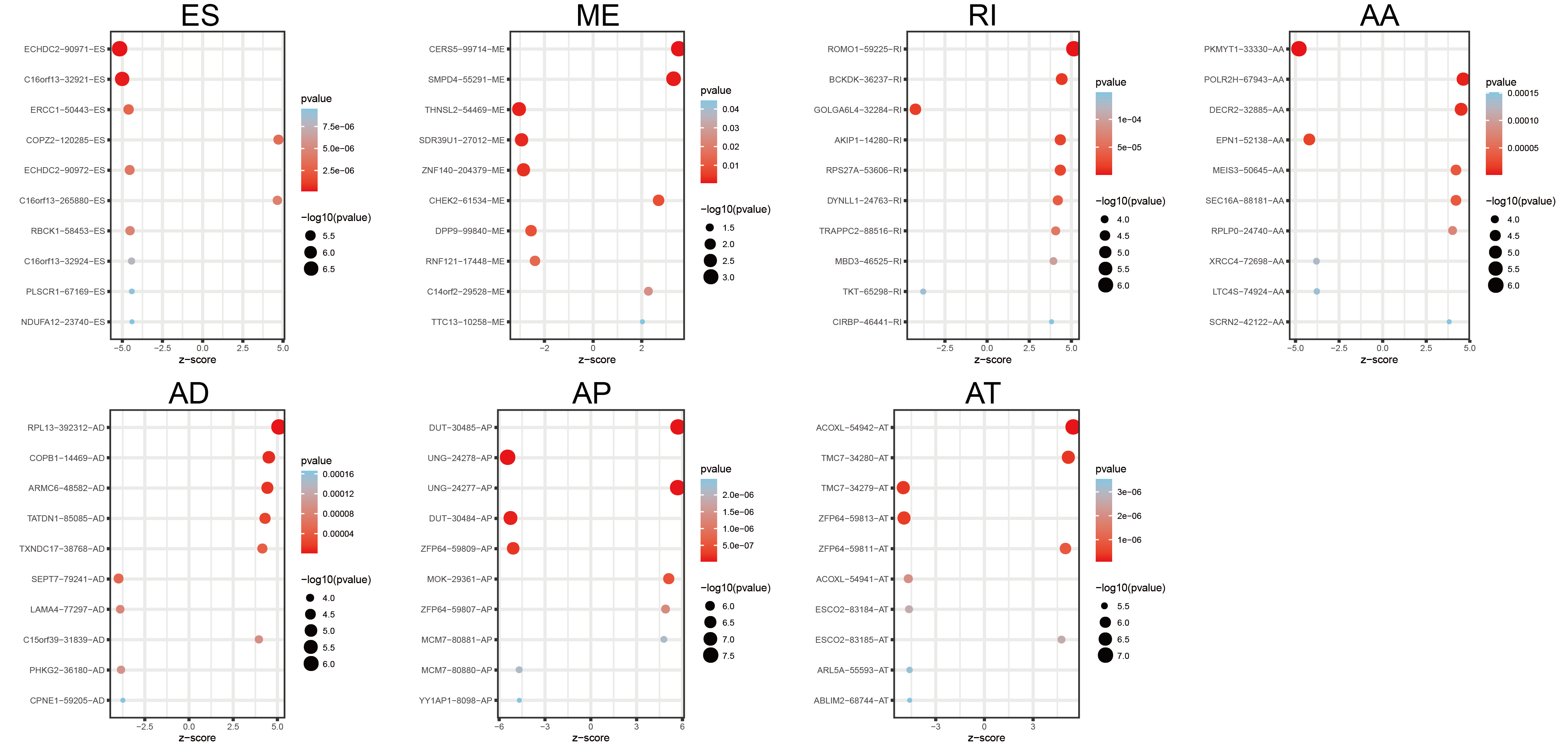


**Fig. S1**: Bubble plots of the top 10 significant survival-related AS events.

Fig. S2


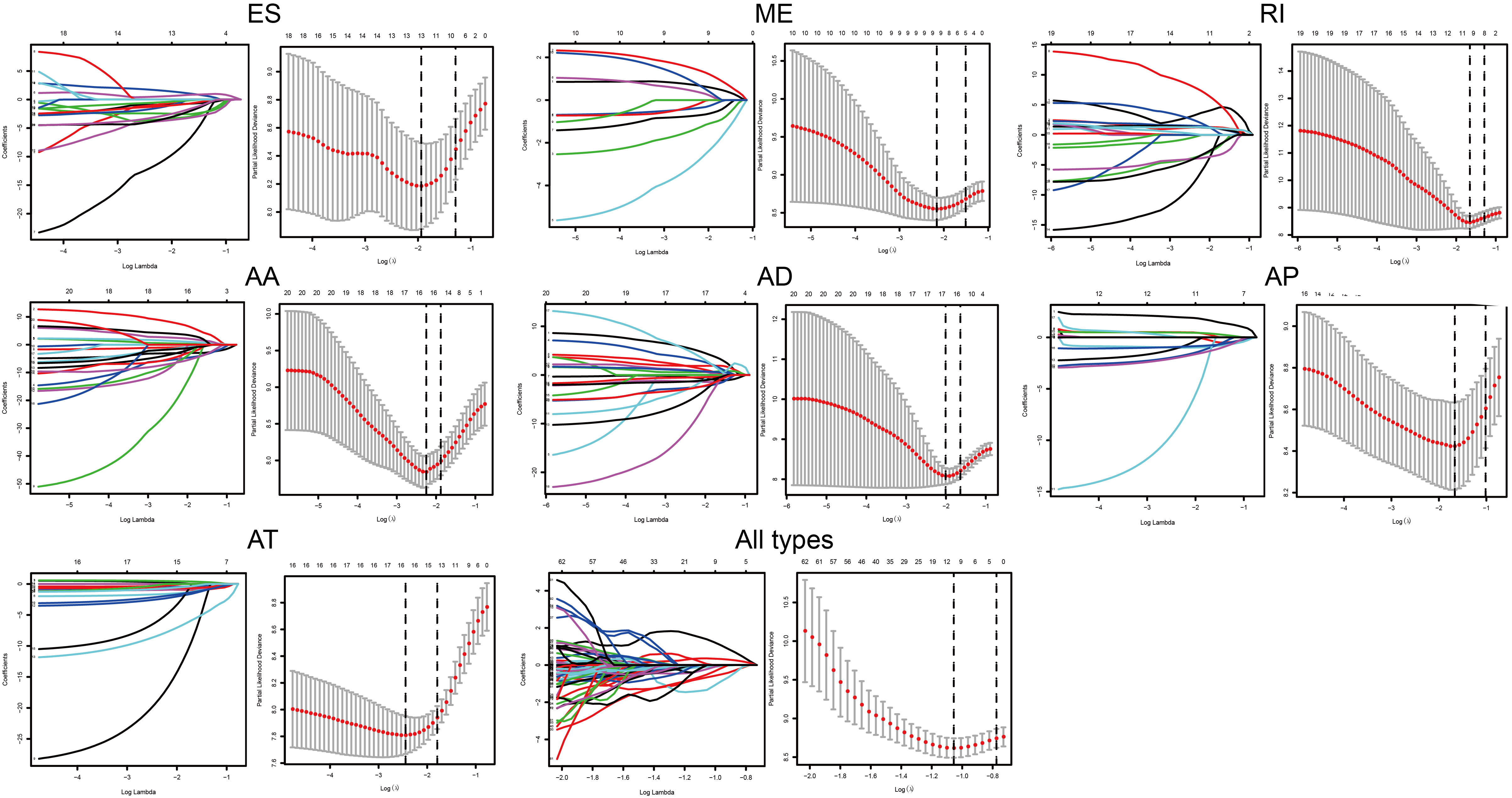


**Fig. S2:** LASSO regression analysis was used to filter the modeling AS events from 3976 survival-related AS events.

Fig.S3


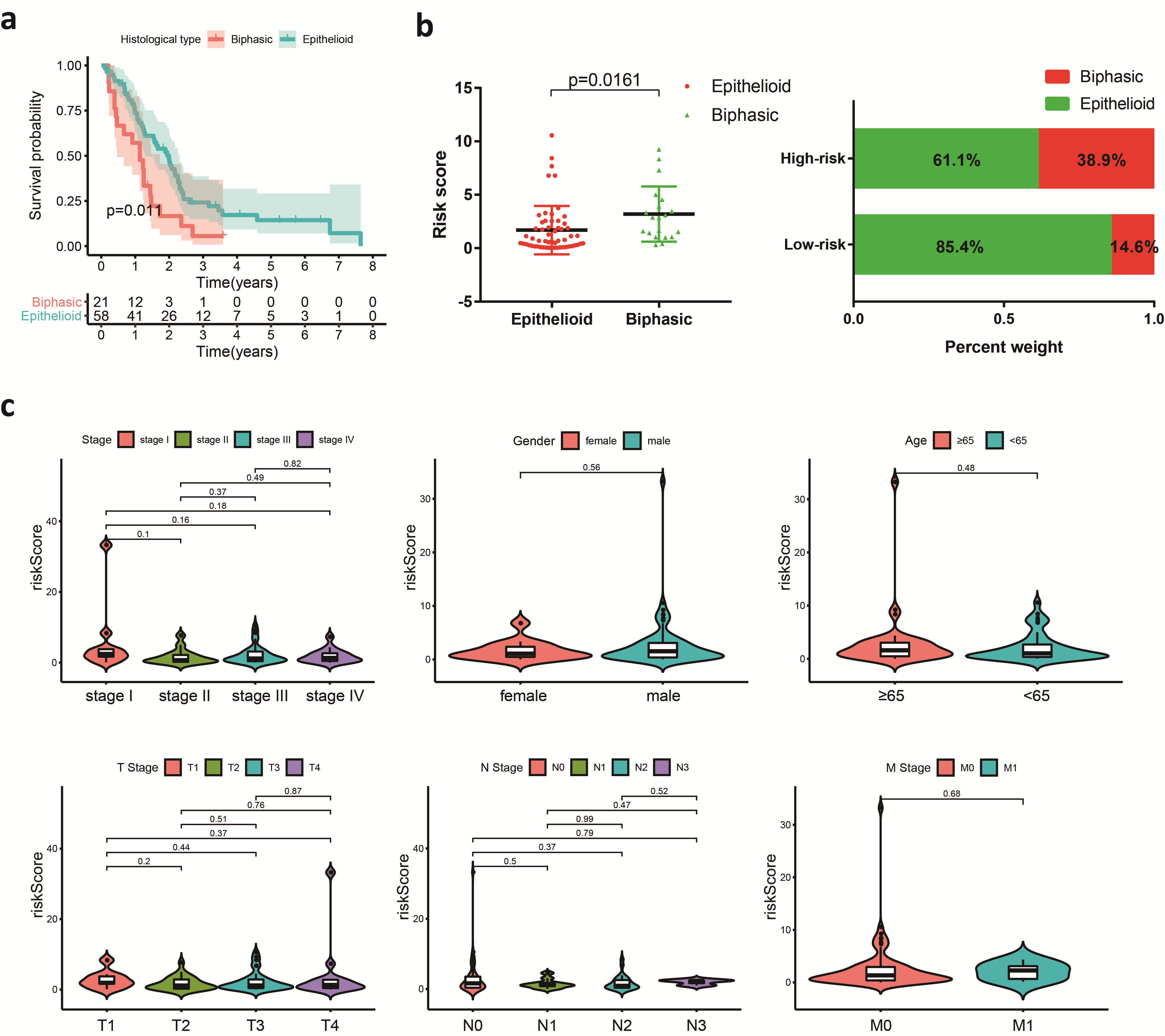


**Fig. S3:** **a** Kaplan-Meier survival analysis of OS between epithelioid and biphasic subtype in MM patients. **b** (Left) The distribution of risk scores between epithelioid and biphasic subtypes. (Right) The distribution of histologic subtypes between low-risk and high-risk groups. **c** The violin plot of correlation between risk score and clinical characteristics including clinical stage, gender, age, T stage, N stage and M stage.

Fig.S4


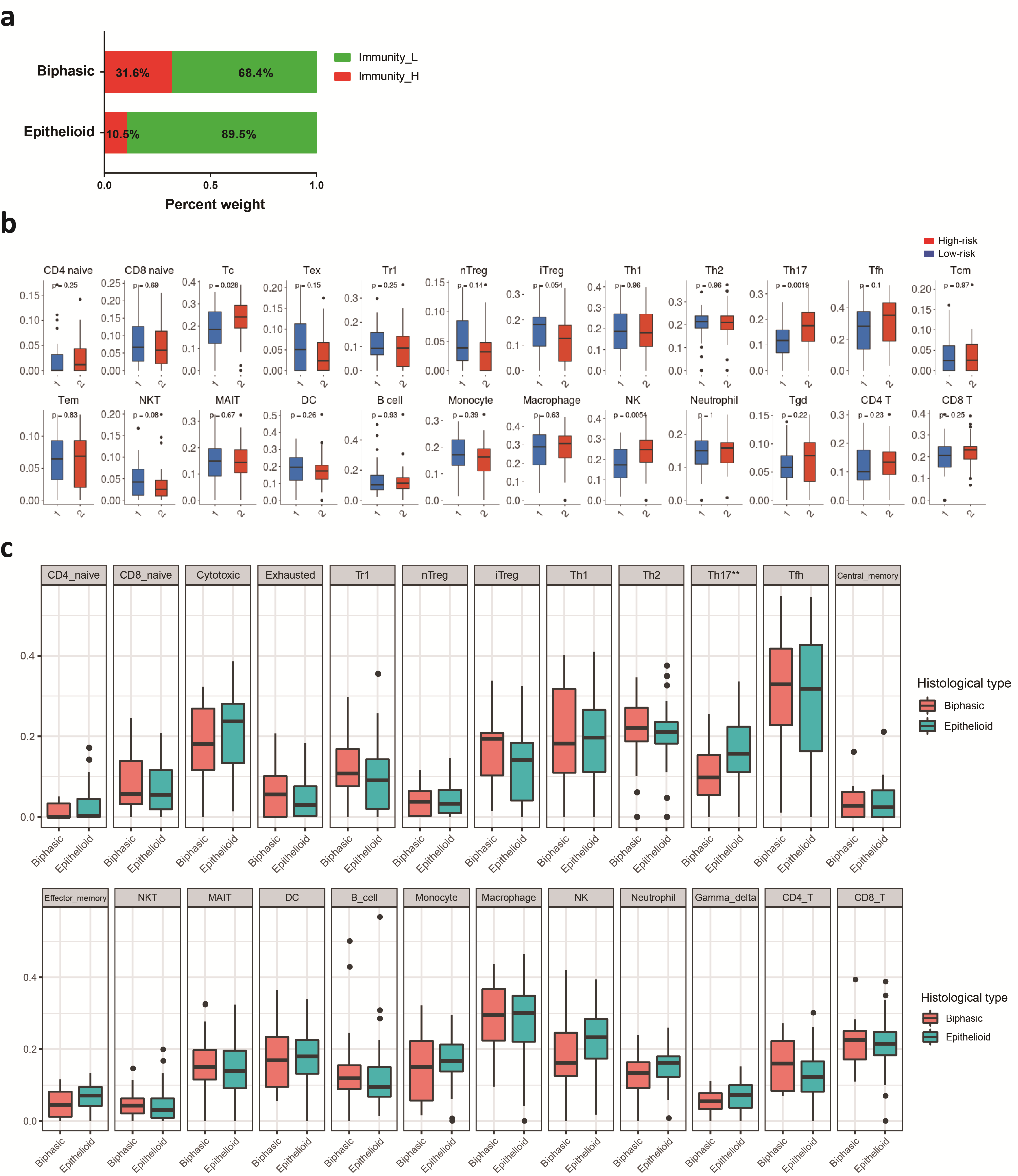


**Fig. S4:** **a** The distribution of immune infiltration level between epithelioid and biphasic subtypes. **b** Comparisons of the abundances of 24 types of tumor-infiltrating immune cells between low-risk and high-risk groups. **c** Comparisons of the abundances of 24 types of tumor-infiltrating immune cells between epithelioid and biphasic subtypes.

Fig.S5


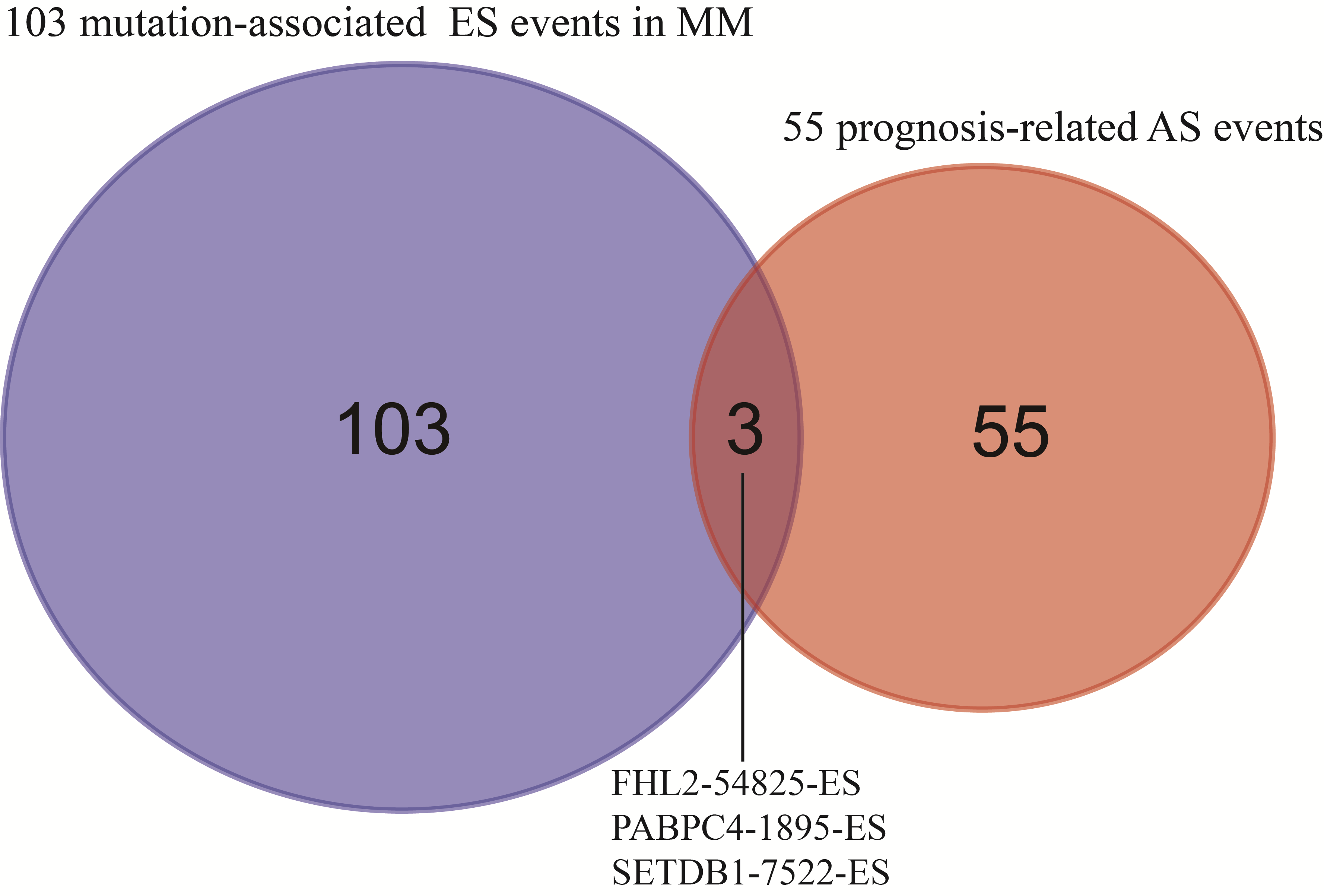


**Fig. S5:** The Venn plot to identify the overlapped genes related to parent genes of 55 prognosis-related AS events and 103 mutation-associated ES events in MM.
